# Elevated CO_2_ Increases Nitrogen Fixation at the Reproductive Phase Contributing to Various Yield Responses of Soybean Cultivars

**DOI:** 10.3389/fpls.2017.01546

**Published:** 2017-09-14

**Authors:** Yansheng Li, Zhenhua Yu, Xiaobing Liu, Ulrike Mathesius, Guanghua Wang, Caixian Tang, Junjiang Wu, Judong Liu, Shaoqing Zhang, Jian Jin

**Affiliations:** ^1^Key Laboratory of Mollisols Agroecology, Northeast Institute of Geography and Agroecology, Chinese Academy of Sciences Harbin, China; ^2^Division of Plant Science, Research School of Biology, Australian National University Canberra, ACT, Australia; ^3^Centre for AgriBioscience, La Trobe University Bundoora, VIC, Australia; ^4^Key Laboratory of Soybean Cultivation of Ministry of Agriculture, Soybean Research Institute, Heilongjiang Academy of Agricultural Sciences Harbin, China

**Keywords:** open-top chamber, ^15^N labeling, nodule density, symbiotic N_2_ fixation, N remobilization, *Glycine max* L.

## Abstract

Nitrogen deficiency limits crop performance under elevated CO_2_ (eCO_2_), depending on the ability of plant N uptake. However, the dynamics and redistribution of N_2_ fixation, and fertilizer and soil N use in legumes under eCO_2_ have been little studied. Such an investigation is essential to improve the adaptability of legumes to climate change. We took advantage of genotype-specific responses of soybean to increased CO_2_ to test which N-uptake phenotypes are most strongly related to enhanced yield. Eight soybean cultivars were grown in open-top chambers with either 390 ppm (aCO_2_) or 550 ppm CO_2_ (eCO_2_). The plants were supplied with 100 mg N kg^−1^ soil as ^15^N-labeled calcium nitrate, and harvested at the initial seed-filling (R5) and full-mature (R8) stages. Increased yield in response to eCO_2_ correlated highly (*r* = 0.95) with an increase in symbiotically fixed N during the R5 to R8 stage. In contrast, eCO_2_ only led to small increases in the uptake of fertilizer-derived and soil-derived N during R5 to R8, and these increases did not correlate with enhanced yield. Elevated CO_2_ also decreased the proportion of seed N redistributed from shoot to seeds, and this decrease strongly correlated with increased yield. Moreover, the total N uptake was associated with increases in fixed-N per nodule in response to eCO_2_, but not with changes in nodule biomass, nodule density, or root length.

## Introduction

Plant demand for nitrogen (N) likely increases under elevated atmospheric CO_2_ (eCO_2_). Nitrogen addition enhances CO_2_ effects on plant productivity. In ryegrass swards, compared to non-N control, N addition resulted in a greater yield response to eCO_2_ (Schneider et al., 2004). Moreover, eCO_2_ significantly increased N uptake of wheat (Butterly et al., 2016). It appears that sufficient N supply may lead to optimization of photosynthetic processes to favor the productivity under eCO_2_ (Ainsworth and Long, 2005; Luo et al., 2006; Langley and Megonigal, 2010).

Therefore, the magnitude of response in plant productivity largely depends on how plant N uptake is capable to keep pace with eCO_2_-induced stimulation of carbohydrate production and growth. Plants may positively regulate a series of physiological processes, such as secretion of enzymes and root growth, to increase the capacity of plant nutrient acquisition for optimal adaptability to eCO_2_ (Rogers et al., 2006; Sardans and Peñuelas, 2012). In legumes, symbiotic N_2_ fixation has been considered as the most influential factor affecting plant N uptake and productivity under eCO_2_ (Ainsworth et al., 2003). Elevated CO_2_ increased nodule size and number, specific nitrogenase activity and plant N content, and consequently increased biomass and/or seed yield in legumes such as *Trifolium repens, Lupinus albus, Pisum sativum*, and *Glycine max* (Zanetti et al., 1996, 1997; Lee et al., 2003; Rogers et al., 2009; Butterly et al., 2016). However, the responses of symbiotic N_2_ fixation to eCO_2_ may vary between legume species and even varieties within a given species. For example, Lam et al. (2012) reported that eCO_2_ (550 ppm) significantly increased the amount of symbiotic N_2_ fixation in the soybean (*G. max*) cultivar Zhonghuang 13 but had no effect in Zhonghuang 35.

Labile N in soil is an important source to satisfy plant N demand under eCO_2_ (Shimono and Bunce, 2009). Studies have shown that the increased root biomass of crops grown under eCO_2_ could increase N uptake from soil (Matamala and Schlesinger, 2000; Bertrand et al., 2007). Moreover, Matamala et al. (2003) reported that under eCO_2_, fine roots are more important for N uptake than total root biomass. However, to our knowledge, the extent of N originating from N_2_ fixation and soil/fertilizer among the soybean cultivars in response to eCO_2_ has not been quantified, especially in Mollisol regions where soybean is a major crop (Liu and Herbert, 2002; Yu et al., 2016). Investigating the cultivar variation in N uptake in response to eCO_2_ is essential to predict the adaptability of soybean cultivars and formulate the N fertilization strategy to increase N-use efficiency in the future.

Besides plant N uptake, the remobilization of N from vegetative to reproductive sinks during the reproductive stages of crop development is an important contributor to maximizing yield in soybean. Because N previously accumulated in vegetative organs can be remobilized to seeds when exogenous N cannot fulfill the N demand in seed filling (Salon et al., 2001; Schiltz et al., 2005), the effect of eCO_2_ on the dynamics of N accumulation might determine the pattern of N remobilization. It has been reported that the extent of the contribution of N remobilization to seed N varies from 80 to 90% in soybean cultivars (Warembourg and Fernandez, 1985; Kinugasa et al., 2012). However, few studies have investigated the N remobilization of soybean cultivars in response to eCO_2_.

Therefore, N uptake and its partitioning in plants under eCO_2_ are important characteristics of phenotypic plasticity in response to climate change. While most previous studies have focused on responses in single genotypes, or compared different unrelated species, our study utilized a group of soybean genotypes that differed in their plastic responses to eCO_2_. Using the ^15^N dilution method (Unkovich and Baldock, 2008), we aimed to assess the effect of eCO_2_ on the origins of plant N, i.e., symbiotically fixed-N, fertilizer N, and soil N, and the correspondent N remobilization during the seed-filling stage. We then correlated these changes with yield stimulation under eCO_2_. We hypothesized that eCO_2_ would increase N_2_ fixation and alter distribution of the fixed-N to seed to contribute to yield gain.

## Materials and methods

### Research site and experimental design

A pot experiment was conducted in open-top chambers (OTC) at the Northeast Institute of Geography and Agroecology (45°73′N, 126°61′E), Chinese Academy of Sciences, Harbin, China. The experiment had a random block design comprising two atmospheric CO_2_ concentration levels and eight soybean cultivars with three replications. The two CO_2_ levels were ambient CO_2_ (aCO_2_; 390 ppm) and eCO_2_ (550 ppm). Each couple of OTC (one per CO_2_ treatment) was considered as a block, and they were randomly located in the field site. The eight soybean cultivars were Xiaohuangjin (XHJ, released in 1951), Hejiao 6 (HJ6, released in 1962), Nenfeng 1 (NF1, released in 1972), Nenfeng 9 (NF9, released in 1980), Suinong 8 (SN8, released in 1989), Suinong 14 (SN14, released in 1996), Heinong 45 (HN45, released in 2003), Suinong 22 (SN22, released in 2005). These cultivars have been widely grown in northeast China with a growing area of more than 2 million ha (Jin et al., 2012).

Six octagonal OTC (three for each CO_2_ concentration) were constructed with a steel frame. The main body of each OTC is 3.5 m in diameter, 2.0 m high and with a 0.5-m high canopy, which formed a 45° angle with the plane (Zhang et al., 2014). The OTC were covered with polyethylene film (transparency ≥95%). This OTC design has been widely used in CO_2_-associated studies (e.g., Liu et al., 2016; Yu et al., 2016; Chaturvedi et al., 2017). A digital CO_2_-regulating system (Beijing VK2010, China) was installed to monitor the CO_2_ level in each OTC and automatically regulate the supply of CO_2_ gas (99.9%) to achieve CO_2_ concentrations of 550 ± 30 ppm for eCO_2_ and 390 ± 30 ppm for aCO_2_. There were 16 pots per OTC with two pots per cultivar for two harvest time points.

### Plant growth and ^15^N labeling

The soil used in this study was classified as a Mollisol, and had an organic C content of 28.3 mg g^−1^ soil, total N of 2.24 mg g^−1^ soil, available N of 260 μg g^−1^ soil, and a pH of 6.97 (1:5 H_2_O). Nitrogen fertilizer was applied as Ca(NO_3_)_2_ with 5% of ^15^N atom excess at a rate of 100 mg N kg^−1^ soil. The procedure of ^15^N labeling is described in Li et al. (2016).

Before sowing, uniform seeds were selected and germinated at 25°C on moistened filter paper. After 2-day germination, six seeds were sown in each pot (20 cm diameter and 40 cm high) containing 9 L soil and thinned to 2 plants 10 days after emergence. Thus, there were six pots per cultivar grown in either aCO_2_ or eCO_2_ environment. The pot design was considered appropriate for precise isotope labeling and root sampling (Ainsworth et al., 2002). However, the pot size used in this experiment might limit, to some extent, the plant response to CO_2_ elevation as Arp (1991) stated that plants grown in pots of 3.5–12.5 L had intermediate responses to eCO_2_. Soil water content was maintained at 80 ± 5% of field capacity by weighing and watering. In addition, wheat (*Triticum aestivum* L. cv. Longmai 26) plants were grown under the same conditions as non-N_2_ fixing reference species (Rennie and Dubetz, 1986) due to lack of suitable non-nodulating isolines, and was harvested at physiological maturity. Although choosing wheat as non-fixing control exhibits some methodological limitations (Unkovich and Baldock, 2008), wheat has been widely used as a reference plant species in many studies to estimate legume N_2_ fixation (Rennie and Dubetz, 1986; Carranca et al., 1999; Lam et al., 2012).

### Harvest and measurements

Plants of three pots were harvested at the R5 (beginning seed formation, 81 days after sowing) and R8 stages (maturity, 120 days after sowing), respectively (Fehr et al., 1971). Shoots were cut at the cotyledon node level and separated into stems plus petioles, leaves and pods at R5, and additionally seeds at R8. The abscised leaves in each pot between R5 and R8 stages were collected for C and N measurements. The entire root system of each plant was carefully separated from soil, and then washed with tap water to remove soil particles adhering to the roots. Nodules were removed from the root system, counted and weighed. The root length and diameter classes of roots were then determined using WinRhizo 2004b (Régent Instruments Inc., Québec, Canada). According to their diameter, roots were classified as fine roots (<0.5 mm), intermediate roots (0.5–1.0 mm), and coarse roots (>1 mm) (Costa et al., 2002).

All plant samples were dried at 70°C for 72 h, and then finely ground in a ball mill (Retsol MM2000, Retsch, Haan, Germany). The ^15^N/^14^N ratio of all samples was measured with an isotope ratio mass spectrometer (Delta^plus^, Finnigan MAT GmbH, Bremen, Germany). The C and N contents of plant samples were determined using an ELEMENTAR III analyzer (Hanau, Germany).

### Calculations and statistical analysis

Atom% ^15^N excess was calculated with reference to the natural ^15^N abundance in the atmosphere (0.3663 atom% ^15^N; Mariotti et al., 1984). The percentage of plant N derived from N_2_ fixation (%Ndfa) was calculated as follows (Rennie and Dubetz, 1986):

%Ndfa = {1 − [atom% ^15^N excess (*fs*)/atom% ^15^N excess (*nfs*)]} × 100

where *fs* and *nfs* represented fixing and non-fixing (wheat) system, respectively.

N_2_ fixed was calculated as follows:

N_2_ fixed (mg plant^−1^) = (%Ndfa/100) × N_plant_(mg plant^−1^)

where N_plant_ was the N content of each plant compartment.

The amounts of plant N derived from fertilizer (Ndff_plant_) and soil (Ndfs_plant_) were estimated (Martínez-Alcántara et al., 2012) as follows:

Ndff_plant_ = N_plant_(mg plant^−1^) × N atom% ^15^N excess in plant/N atom%

^15^N excess in fertilizer (19.83%)

Ndfs_plant_ = N_plant_(mg plant^−1^) − Ndff_plant_ − N_2_ fixed

The amount of N remobilized from vegetative organs to seeds was estimated as N content in vegetative organs aboveground at R5 subtracted from that at R8 (Egli et al., 1978). Nodule density was calculated as nodule number divided by total root length. Two-way ANOVA on variables including yield components, parameters of plant N, root morphology, nodule number, and nodule fresh weight was performed with Genstat 13 (VSN International, Hemel Hemspstead, UK). Partial correlation analyses were used to evaluate the correlations of N assimilation indices with nodule characteristics, root morphology and yield gain in response to eCO_2_ (Peng et al., 2004). The least significance difference (LSD) was used to assess the differences among treatments at *P* = 0.05.

## Results

### Seed yield and seed N origins

**Figure 1:**
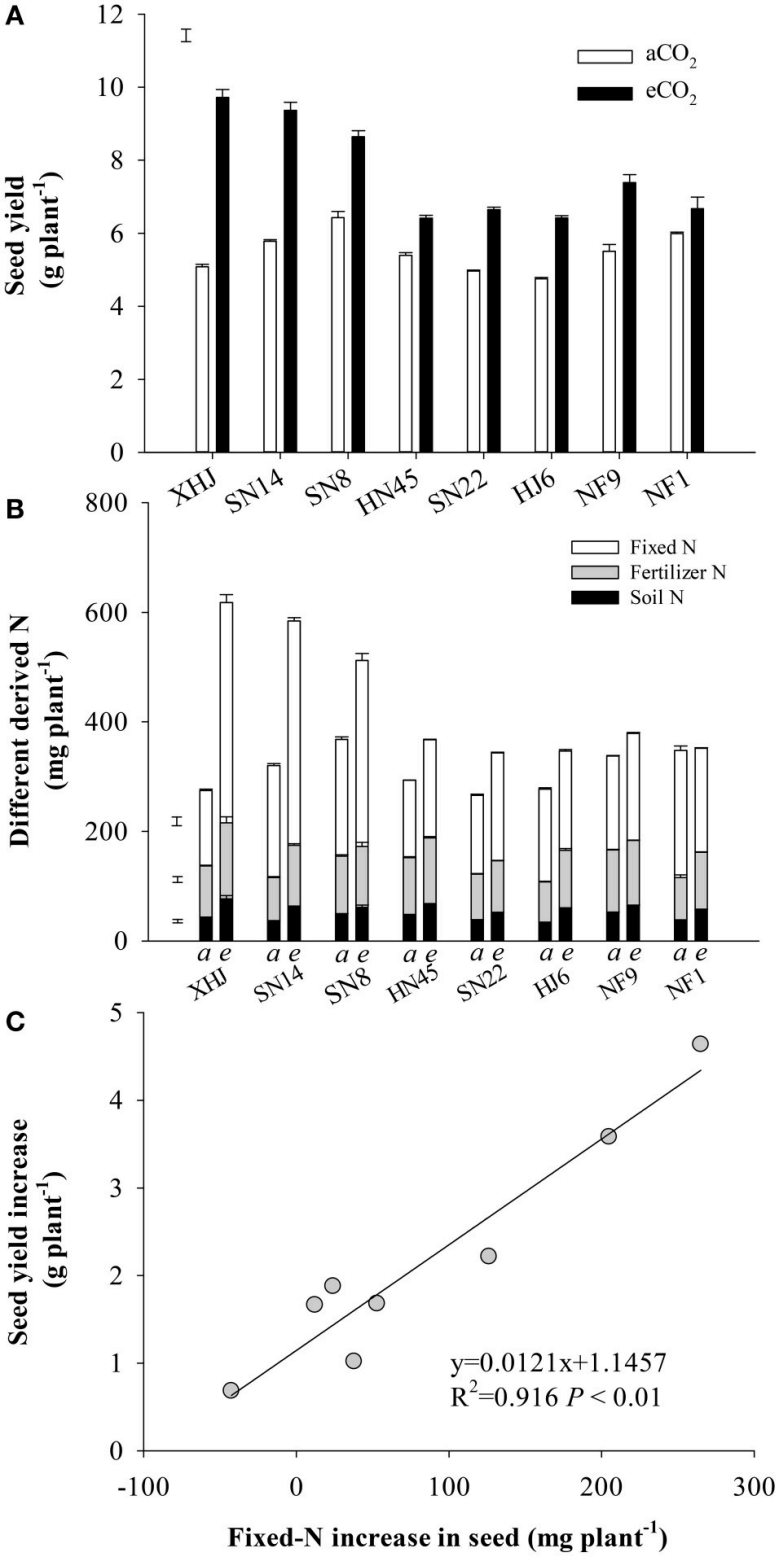
**(A)** Seed yield, **(B)** soil-derived N, fertilizer-derived N, and symbiotically fixed-N content in seed and **(C)** the relationship between increased fixed-N in seeds and yield increment of eight soybean cultivars under eCO_2_ relative to aCO_2_. Each data point represents one cultivar. The error bars represent standard error, and separate vertical bar(s) in **(A)** and **(B)** indicate the LSD (*P* < 0.05) for the CO_2_ × cultivar interaction.

Compared to aCO_2_, eCO_2_ increased seed yield by an average of 40% (Figure 1A). The yield response to eCO_2_ varied among cultivars (*P* < 0.001), resulting in a 91% increase in XHJ in comparison to 12% in NF1, and leading to a significant CO_2_× cultivar interaction (*P* < 0.001). Interestingly, the cultivars showing the highest yield under eCO_2_ were not the ones showing the highest yield under aCO_2_, but exhibited the biggest increase in yield gain. In addition, the N content of the seed showed a shift in origin toward greater fixed N under eCO_2_ (Figure 1B). Overall, there was a strong (*P* < 0.001) correlation between the increase in fixed-N content of seeds and their yield increase under eCO_2_ (Figure 1C).

### Shoot biomass and N content

Shoot biomass at R8 also significantly increased (by 46% on average) under eCO_2_ compared with aCO_2_ (Figure S1) with a minimum increase of 22% for HN45 and a maximum of 87% for XHJ (*P* < 0.001). Compared with aCO_2_, eCO_2_ increased shoot N content by 11% at R5, and 41% at R8 (*P* < 0.05) (Table 1). Among cultivars, the largest increase in shoot N content at R8 in response to eCO_2_ was observed in XHJ (119%), and the smallest one (7%) in NF1.

**Table 1:** Shoot N content, symbiotically fixed-N (SNF) content, fertilizer-derived, and soil-derived N content in shoot of eight soybean cultivars grown under aCO_2_ or eCO_2_ till R5 (81 days after sowing) and R8 (120 days after sowing).

	**N content (mg plant**^**−1**^**)**		**SNF N content (mg plant**^**−1**^**)**		**Fertilizer N content (mg plant**^**−1**^**)**		**Soil N content (mg plant**^**−1**^**)**	
	**aCO_2_**	**eCO_2_**	**aCO_2_**	**eCO_2_**	**aCO_2_**	**eCO_2_**	**aCO_2_**	**eCO_2_**
**R5 (81 DAYS AFTER SOWING)**								
XHJ	220	262^*^	79.0	79.2^ns^	96.6	118^*^	44.4	64.8^*^
SN14	216	233^ns^	80.5	64.6^*^	93.4	107^*^	42.9	58.9^*^
SN8	240	266^ns^	89.9	59.3^*^	115	118^ns^	52.9	65.2^*^
HN45	258	243^*^	74.5	62.5^*^	95.1	112^*^	43.7	61.7^*^
SN22	217	231^*^	78.8	56.4^*^	79.1	106^*^	36.4	58.7^*^
HJ6	218	264^*^	93.5	80.7^*^	85.3	118^*^	39.2	65.0^*^
NF9	213	236^*^	83.3	67.6^*^	107	128^*^	49.2	70.5^*^
NF1	194	222^ns^	76.6	61.2^*^	95.5	111^*^	43.9	61.2^*^
LSD_0.05_	19.6		7.76		9.16		4.64	
**SIGNIFICANCE LEVEL**								
CO_2_	<0.001		<0.001		<0.001		<0.001	
Cultivar	<0.001		<0.001		<0.001		<0.001	
CO_2_×Cultivar	0.004		<0.001		0.004		0.009	
**R8 (120 DAYS AFTER SOWING)**								
XHJ	339	742^*^	162	466^*^	114	167^*^	63.6	110^*^
SN14	405	680^*^	249	461^*^	97.1	127^*^	59.1	91.6^*^
SN8	448	626^*^	248	399^*^	126	134^ns^	73.7	93.9^*^
HN45	356	461^*^	174	233^*^	116	133	66.5	94.7^*^
SN22	329	423^*^	169	231^*^	102	112^*^	58.3	79.0^*^
HJ6	349	438^*^	201	223^*^	91.8	125^*^	55.5	90.0^*^
NF9	418	466^*^	215	241^*^	128	130^ns^	74.7	94.8^*^
NF1	415	442^*^	261	234^ns^	95.9	121^*^	57.8	87.6^*^
LSD_0.05_	22.4		16.0		12.4		7.31	
**SIGNIFICANCE LEVEL**								
CO_2_	<0.001		<0.001		<0.001		<0.001	
Cultivar	<0.001		<0.001		<0.001		<0.001	
CO_2_×Cultivar	<0.001		<0.001		<0.001		<0.001	

* *and ns indicate significant and non-significant difference (t-test) between aCO_2_ and eCO_2_, respectively, for individual cultivars. LSD values correspond to the CO_2_ × cultivar interaction (two-way ANOVA)*.

Elevated CO_2_ decreased shoot N concentration (mg g^−1^) by an average of 30% at R5 (Figure S1). At R8, eCO_2_ did not affect shoot N concentration in SN8, SN14, HN45, and SN22 (Figure S1), but increased it by 17% in XHJ.

### Shoot N origins

Compared to aCO_2_, eCO_2_ decreased the fixed-N content (mg plant^−1^) of the shoot at R5 (*P* < 0.05), but significantly increased it at R8 (Table 1). The maximum increase was found in XHJ (188%) while no difference occurred in NF1 (*P* > 0.05) at R8 (Table 1).

Elevated CO_2_ increased the accumulation of the fertilizer-derived N in the shoot (mg plant^−1^) by 20 and 21% at R5 and R8, respectively (Table 1). The extent of increase of fertilizer-derived N under eCO_2_ differed among cultivars. At R5, the increase in fertilizer-derived N in HJ6 under eCO_2_ reached 38% compared to aCO_2_, while there was no CO_2_ effect in SN8. At R8, eCO_2_ increased fertilizer-derived N by 46% in XHJ but did not affect it in SN8 and NF9. A significant (*P* < 0.001) CO_2_× cultivar interaction was observed at R5 and R8 (Table 1).

Similarly, eCO_2_ increased the soil-derived N accumulation in the shoot by 45 and 47% at R5 and R8, respectively (Table 1). A significant CO_2_× cultivar interaction on soil-derived N content in the shoot was observed (Table 1). At R5, soil-derived N content increased by 66% in HJ6 under eCO_2_ in comparison to 23% in SN8. At R8, XHJ exhibited 73% increase for soil-derived N content, but only 27% increase in SN8 and NF1 was observed. However, overall, there was no significant correlation between yield gain and either soil-derived or fertilizer-derived N uptake under eCO_2_ (Figure S2).

**Figure 2:**
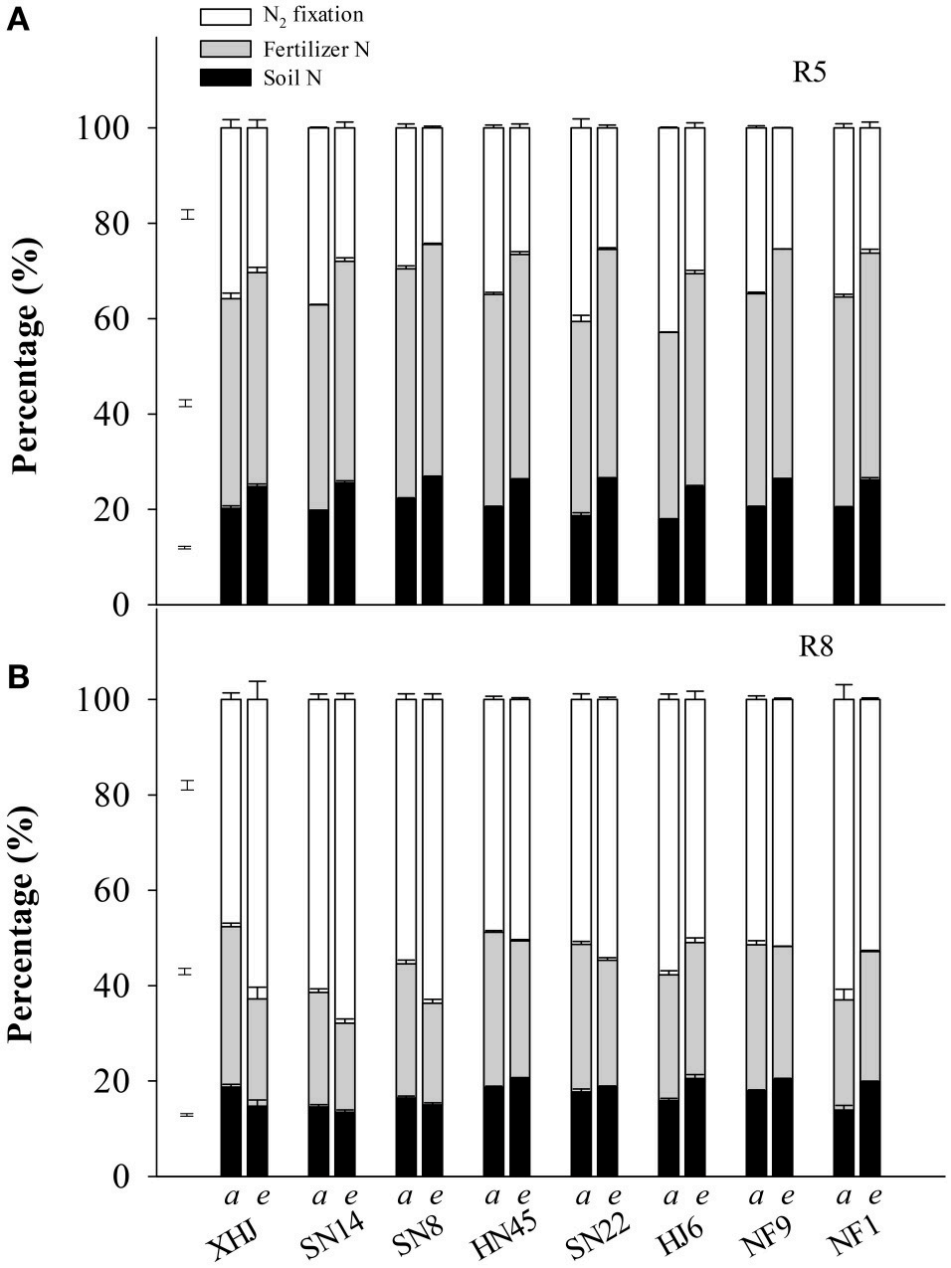
The effect of CO_2_ on the percentage of plant N derived from symbiotically fixed-, fertilizer-, and soil-N at **(A)** R5 and **(B)** R8 (81 and 120 days after sowing, respectively). The error bars represent standard error, and separate vertical bars indicate the LSD (*P* < 0.05) for the CO_2_ × cultivar interaction. The letters of *a* and *e* on the x-axis indicate aCO_2_ and eCO_2_, respectively.

Under eCO_2_, the proportion of fixed-N in the shoot at R5 decreased (*P* < 0.05) by 27% compared to aCO_2_ (Figure 2A). In contrast, the proportion of fertilizer- and soil-derived N in the shoot at R5 increased by 9.1 and 31%, respectively, under eCO_2_. At R8, however, eCO_2_ increased the proportion of fixed-N in the shoot of all cultivars except for HJ6 (−12%) and NF1 (−16%) (Figure 2B). Under eCO_2_, the proportion of fertilizer-derived N decreased in all cultivars. Elevated CO_2_ decreased the proportion of soil-derived N in the shoot of XHJ, SN8, and SN14, but increased it in HJ6, NF1, NF9, HN45, and SN22, leading to significant CO_2_× cultivar interactions (Figure 2B).

### N remobilization

**Figure 3:**
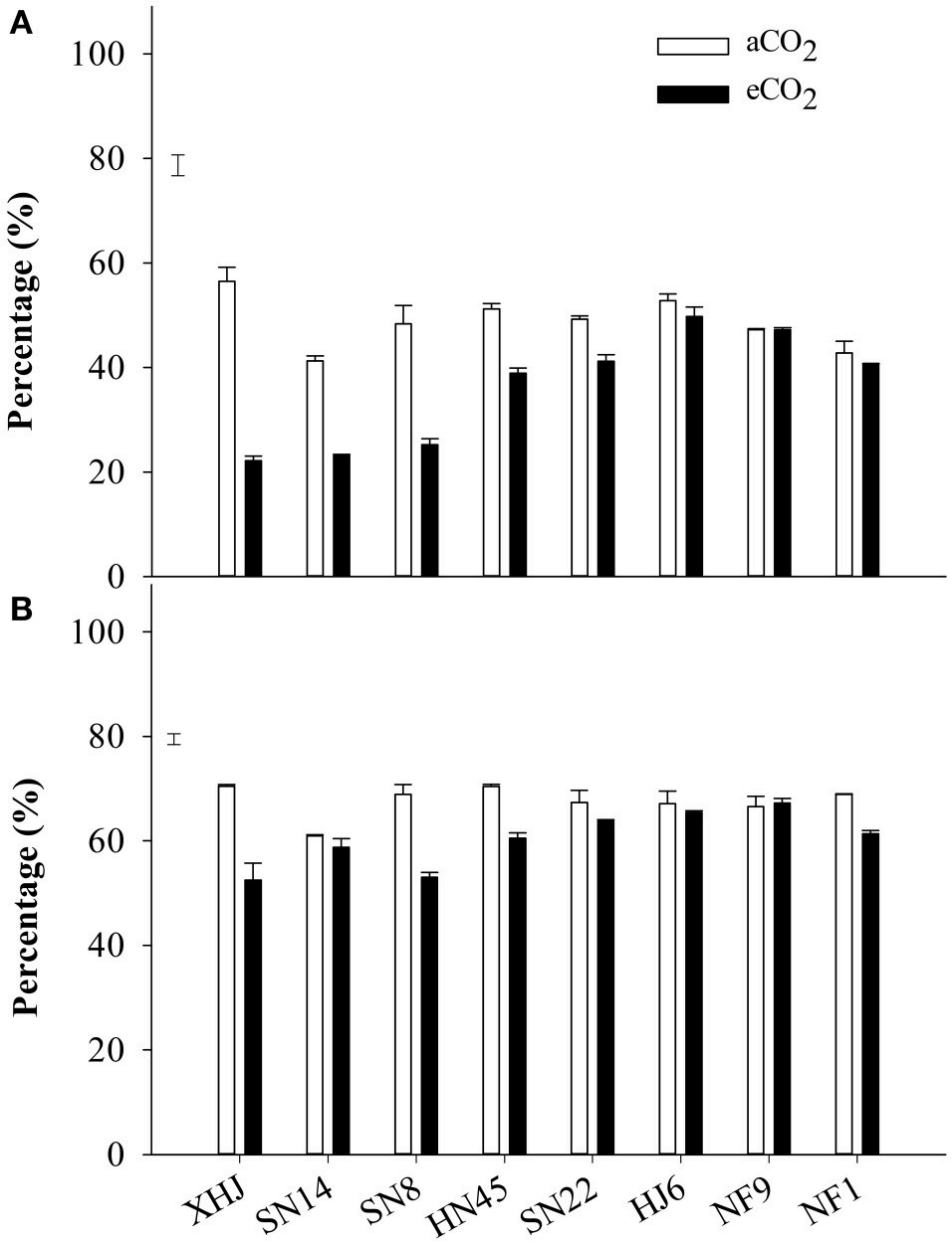
The percentage of **(A)** remobilized N in seed and **(B)** the percentage of N remobilized from vegetative organs to seeds of eight cultivars grown under aCO_2_ or eCO_2_. The error bars represent standard error, and the separate vertical bar in each panel indicates the LSD (*P* < 0.05) for the CO_2_ × cultivar interaction.

Elevated CO_2_ significantly decreased the proportion of the remobilized N in seeds, with the greatest decrease for XHJ and no significant response for HJ6, NF9, and NF1 (Figure 3A).

Approximately 68% of N was remobilized from vegetative organs to seeds at aCO_2_ in comparison to 60% under eCO_2_ (Figure 3B). Elevated CO_2_ significantly (*P* < 0.05) decreased the proportion of the N remobilization in XHJ, NF1, SN8, and HF45, but did not affect it in HJ6, NF9, SN14, and SN22, contributing to a significant CO_2_× cultivar interaction.

### Relationship between yield and N

**Figure 4:**
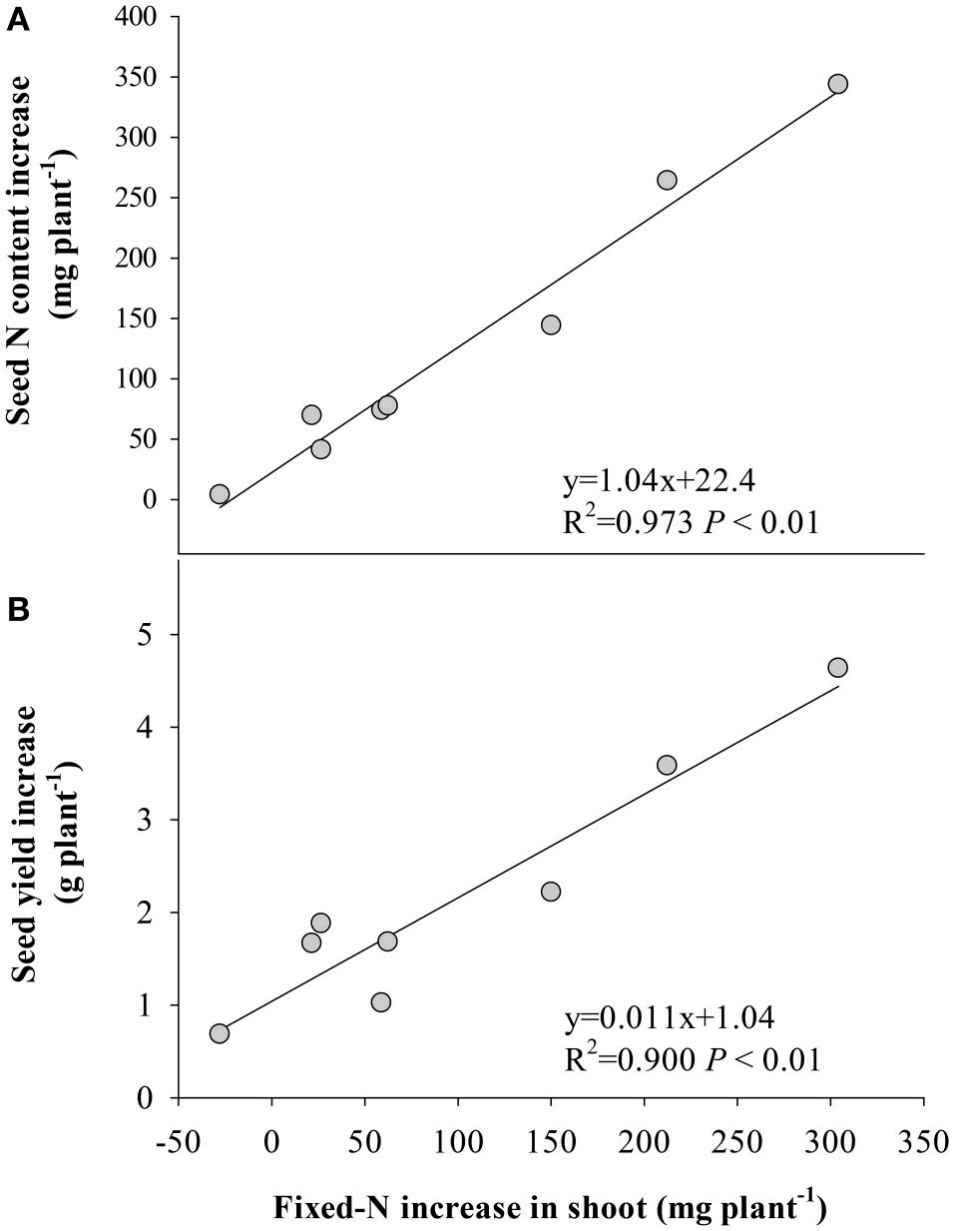
Relationships between the increase in the amount of fixed-N in shoot at R8 (120 days after sowing) and increases in **(A)** seed N and **(B)** seed yield of the eight soybean cultivars under eCO_2_ relative to aCO_2_. Each point represents one cultivar.

**Figure 5:**
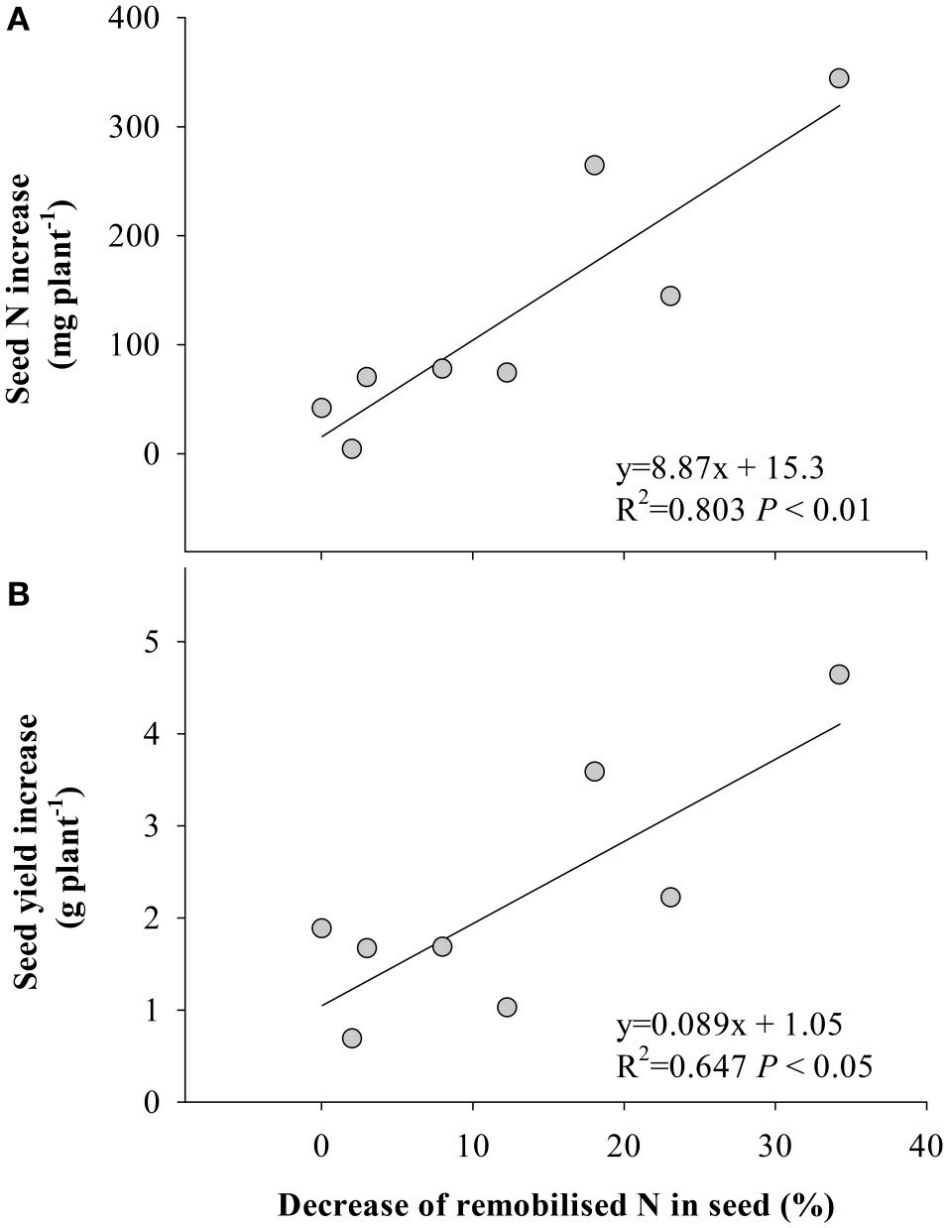
Relationships between the decrease of remobilized N in seed under eCO_2_ and **(A)** the seed N content increase, and **(B)** seed yield increase. Each point represents one soybean cultivar. The decrease of remobilized N was calculated as proportion of remobilized N in seed under aCO_2_ minus that under eCO_2_.

The stimulation of fixed-N was significantly correlated with seed N increase (Figure 4A) and yield gain (Figure 4B), while the decrease of remobilized N to seed significantly correlated with the response of seed N to eCO_2_ (Figure 5A) and yield (Figure 5B). No significant correlation (*P* > 0.05) was found between the increase in fertilizer- or soil-derived N content and the increase of yield in response to eCO_2_ (Figure S3).

### Root morphology

Elevated CO_2_ increased total root length (*P* < 0.05) by an average of 19% (Table S1). The length of fine roots accounted for more than 85% of total root length, and fine roots (<0.5 mm) had a positive (*P* < 0.05) growth response to eCO_2_ in all cultivars except for SN22 (Table S1). Only the length of intermediate roots of XHJ, and the length of coarse roots of SN22 and NF1 were significantly higher under eCO_2_ than under aCO_2_ (*P* < 0.05).

Elevated CO_2_ significantly increased the N uptake per unit of root length in XHJ, SN14, HN45, SN22, and NF1 compared to aCO_2_ (*P* < 0.05), but did not in SN8, HJ6, and NF9 (Table S2). The fertilizer-derived N uptake per unit of root length did not significantly change in response to eCO_2_ except for NF1 (+15%) and NF9 (–12%) (*P* < 0.05). The soil-derived N uptake per unit of root increased by 26% (*P* < 0.05) across the cultivars under eCO_2_ compared to aCO_2_, with the maximum increase (44%) for XHJ and the minimum (9%) for SN8.

Although there were marked changes in root architecture in response to eCO2, these changes did not directly contribute to yield gain under eCO2. There was no correlation between seed yield increase with changes in total root length, fine, intermediate or coarse root length (*P* > 0.05, Figure S4).

### Nodulation

Elevated CO_2_ significantly altered the nodule characteristics of soybean. Nodule numbers increased from 79 under aCO_2_ to 113 under eCO_2_ on average across cultivars (Table 2). Nodule number in response to eCO_2_ differed among soybean cultivars, with 96% of increase in HJ6 in comparison to only 3% in SN14. A significant (*P* < 0.001). A significant CO_2_× cultivars interaction was observed (*P* < 0.001; Table 2). Elevated CO_2_ resulted in a significant increase in nodule fresh weight (Table 2). The maximum increase (301%) was found in SN14 while the minimum increase was 93% in SN22. Elevated CO_2_ significantly increased nodule density of all cultivars but NF9 and SN14 (Table 2).

**Table 2:** Nodule number per plant, nodule fresh weight per plant, nodule density, and single nodule N fixation of eight soybean cultivars grown for 120 days (R8) under aCO_2_ or eCO_2_.

	**Nodule number (number plant**^**−1**^**)**		**Nodule fresh weight (mg plant**^**−1**^**)**		**Nodule density (number m**^**−1**^**)**		**Fixed-N per nodule (mg nodule**^**−1**^**)**	
	**aCO_2_**	**eCO_2_**	**aCO_2_**	**eCO_2_**	**aCO_2_**	**eCO_2_**	**aCO_2_**	**eCO_2_**
XHJ	80	132^*^	368	1,392^*^	1.35	1.87^*^	2.04	3.54^*^
SN14	132	136^ns^	431	1,726^*^	2.71	2.27^*^	1.89	3.42^*^
SN8	78	112^*^	554	1,480^*^	1.19	1.51^*^	3.19	3.58^ns^
HN45	101	142^*^	368	1,035^*^	1.82	2.32^*^	1.73	1.65^ns^
SN22	104	171^*^	712	1,376^*^	1.74	2.54^*^	1.64	1.36^ns^
HJ6	55	108^*^	509	1,698^*^	1.05	1.48^*^	3.71	2.10^*^
NF9	56	62^*^	353	1,125^*^	1.08	1.03^ns^	3.83	3.92^ns^
NF1	30	42^*^	228	560^*^	0.52	0.67^*^	8.97	5.56^*^
LSD_0.05_	10.3		307		0.15		0.33	
**SIGNIFICANCE LEVEL**								
CO_2_	<0.001		<0.001		<0.001		<0.001	
Cultivar	<0.001		<0.001		<0.001		<0.001	
CO_2_×Cultivar	<0.001		0.002		<0.001		<0.001	

* *and ns indicate significant and non-significant difference (t-test) between aCO_2_ and eCO_2_ within a genotype, respectively, for individual cultivars. LSD values correspond to the CO_2_ × cultivar interaction (two-way ANOVA)*.

The amount of N fixed per nodule showed different responses to eCO_2_ among cultivars (Table 2), with 81 and 74% of increase in SN14 and XHJ in comparison to 43 and 38% of reduction in HJ6 and NF1, respectively, resulting in a significant CO_2_× cultivars interaction (*P* < 0.001).

**Figure 6:**
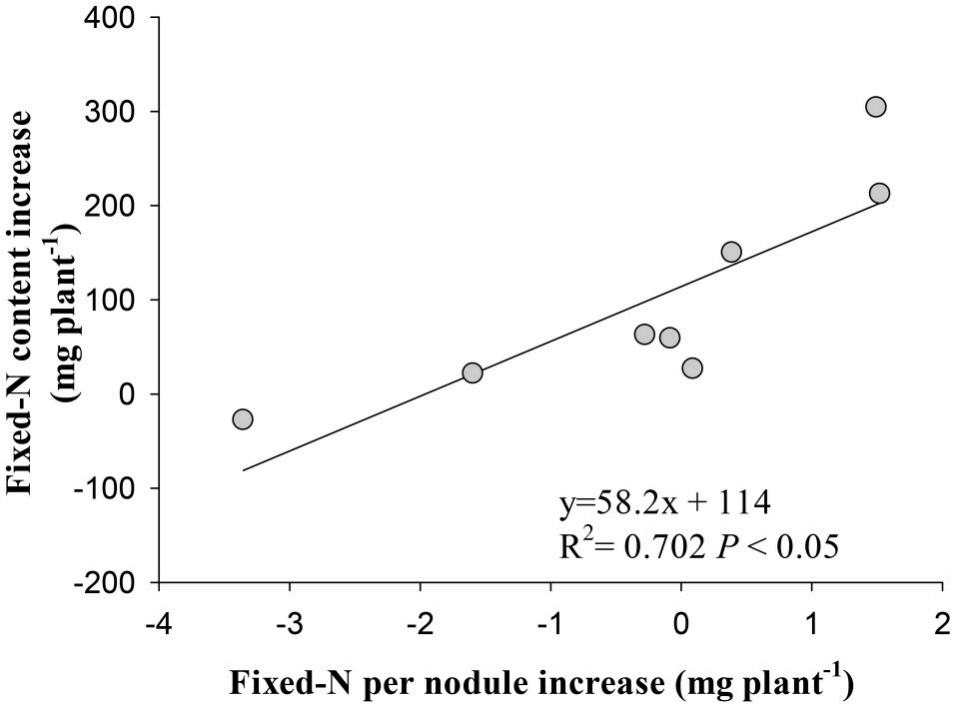
Relationship between the increase in fixed-N per nodule and the increase in fixed-N content in shoot of eight soybean cultivars at R8 (120 days after sowing) under eCO_2_ relative to aCO_2_. Each point represents one cultivar.

Irrespective of cultivars, the increase in symbiotically fixed-N content in shoot correlated positively with the increase of fixed-N per nodule in response to eCO_2_ (*P* < 0.01; Figure 6), but did not correlate with nodule number, fresh weight, and density changes (*P* > 0.05; Figure S4).

## Discussion

This study demonstrated that eCO_2_ enhanced total N uptake in soybean, especially during the late reproductive stages. It was evident that the increase in the N content in shoots under eCO_2_ was greater at R8 than at R5 (Table 1). Moreover, irrespective of cultivars, the extent of the increase in N content derived from symbiotically fixed-N was greater than either fertilizer-derived N or soil-derived N during the period from R5 to R8 (Table 1). The fixed-N was the dominant source of plant N, but the proportion of fixed-N was greater under eCO_2_ than under aCO_2_ (Figure 2). The results are consistent with those of previous studies showing that eCO_2_ increased total N uptake in agricultural crops (Kimball et al., 2002; Leakey et al., 2009; Jin et al., 2012; Lam et al., 2012; Butterly et al., 2016).

Symbiotic N_2_ fixation during this reproductive period is critical for yield gain under eCO_2_. This was supported by the positive correlation (*P* < 0.05) between the amount of symbiotically fixed-N and seed yield (Figure 4), and the fixed-N being the major source of seed N (Figure 1). Furthermore, eCO_2_ decreased the proportion of remobilized N in seed (Figure 3), indicating that the eCO_2_-enhanced total N uptake during the late reproductive stage can largely satisfy N demand in seed development. Since the major source of N remobilization in soybean plants is from leaves (Schiltz et al., 2005; Li et al., 2016), the lesser amount of N removed from vegetative organs including leaves in response to eCO_2_ (Figure 3) was likely to maintain leaf photosynthetic capacity. Makino and Osmond (1991) also showed that leaf N correlated highly with the photosynthetic function of the leaf. Thus, the maintenance of adequate N in vegetative organs is likely to contribute to the increased biomass accumulation and seed yield under eCO_2_ (Figure 5).

The stimulation of N_2_ fixation during R5 to R8 under eCO_2_ was attributed to the increase in nodule N_2_ fixation efficiency, as evidenced by the positive correlation between the increase of fixed-N per nodule with the increase in fixed-N content in shoot under eCO_2_ (Figure 6). In previous studies, eCO_2_ enhanced N_2_ fixation through increasing specific nitrogenase activity (Saeki et al., 2008). The reason for the increased N_2_ fixation is that the enhanced photosynthesis under eCO_2_ (Ziska, 2008; Bishop et al., 2015) provides sufficient C sources for maintaining nodule function and N_2_ fixation (Li et al., 2016), resulting in the increase in shoot and root biomass (Figure S1). Another reason would be a change of rhizobium community in the rhizosphere of soybean under eCO_2_ (Yu et al., 2016), which might favor N_2_ fixation efficiency of nodules. This interaction between functional rhizobia and photosynthetic C supply under eCO_2_ warrants specific investigation.

A number of studies reported that eCO_2_ increased nodule number and biomass in chickpea, field pea (Jin et al., 2012), and common bean (Miyagi et al., 2007; Rogers et al., 2009). In the current study, a similar trend was observed for soybean, but neither the increase of nodule number nor biomass correlated with the increase of fixed-N content (Figure S4). This implies that the increase of fixed-N under eCO_2_ cannot be predominantly attributed to the number of nodules.

Elevated CO_2_ also changed root morphology with an increase in the proliferation of fine roots, which is likely to enhance plant nutrient absorption (Bentley et al., 2013; Beidler et al., 2015). Fine roots play a key role in N acquisition rather than root biomass (Matamala et al., 2003). In this study, the length of fine roots (<0.5 mm) significantly increased under eCO_2_ (Table S1), which helped to increase the uptake of soil and fertilizer N (Table 1). This is consistent with previous studies (Mikan et al., 2000; Zak et al., 2000; de Graaff et al., 2006; Beidler et al., 2015). Rogers et al. (1992) suggested that the greater proliferation of roots grown under eCO_2_ was a strategy to permit adequate nutrient acquisition under sub-optimal water supply. However, compared to fixed-N, the soil-, and fertilizer-derived N in the plant showed much less response to eCO_2_. The increase in fine root growth had no significant correlation with seed yield increase in response to eCO_2_ across genotypes (Figure S3), indicating that the contribution of root N uptake to yield gain is minimal under eCO_2_. In agreement with our observations, Butterly et al. (2015) also found that N fertilizer did not affect plant N concentration, and the proportion of fertilizer-derived N in field pea decreased under eCO_2_.

Nevertheless, eCO_2_ increased the uptake of soil N per unit of root length (Table S2). The enhancement of microbial activity and N mineralization in soil under eCO_2_ might be the main reason. The growth of fine roots leads to more rhizodeposition, which provides labile C for microorganisms to mineralize more soil organic N (Fischer and Kuzyakov, 2010; Fischer et al., 2010).

The capacity for total N uptake in response to eCO_2_ varied among soybean cultivars, XHJ had the greatest increase in N_2_ fixation under eCO_2_ (Figure 2), which supplied a large amount of N to seed during the reproductive stage (Figure 1B), and reduced the demand for N remobilization (Figure 3). In contrast, NF1 did not exhibit any increase in fixed-N during R5 to R8, and had the least increase in yield under eCO_2_ (Figure 1). The largest N_2_ fixation in XHJ would contribute to a high N_2_ fixation efficiency, since the amount of fixed-N per nodule was greatest in this cultivar (Table 2). As the dominant rhizobial strains in nodules greatly affected N_2_-fixing efficiency (Saeki et al., 2008) and soil microbial communities in the rhizosphere in response to eCO_2_ are dependent on soybean cultivars (Yu et al., 2016), the specific interaction between cultivar and rhizobial genera under eCO_2_ may influence soybean adaptability to eCO_2_. Therefore, the cultivar-specific rhizobia community in nodules may predominantly regulate the N_2_-fixing function in response to eCO_2_. This hypothesis deserves further research.

**Figure 7:**
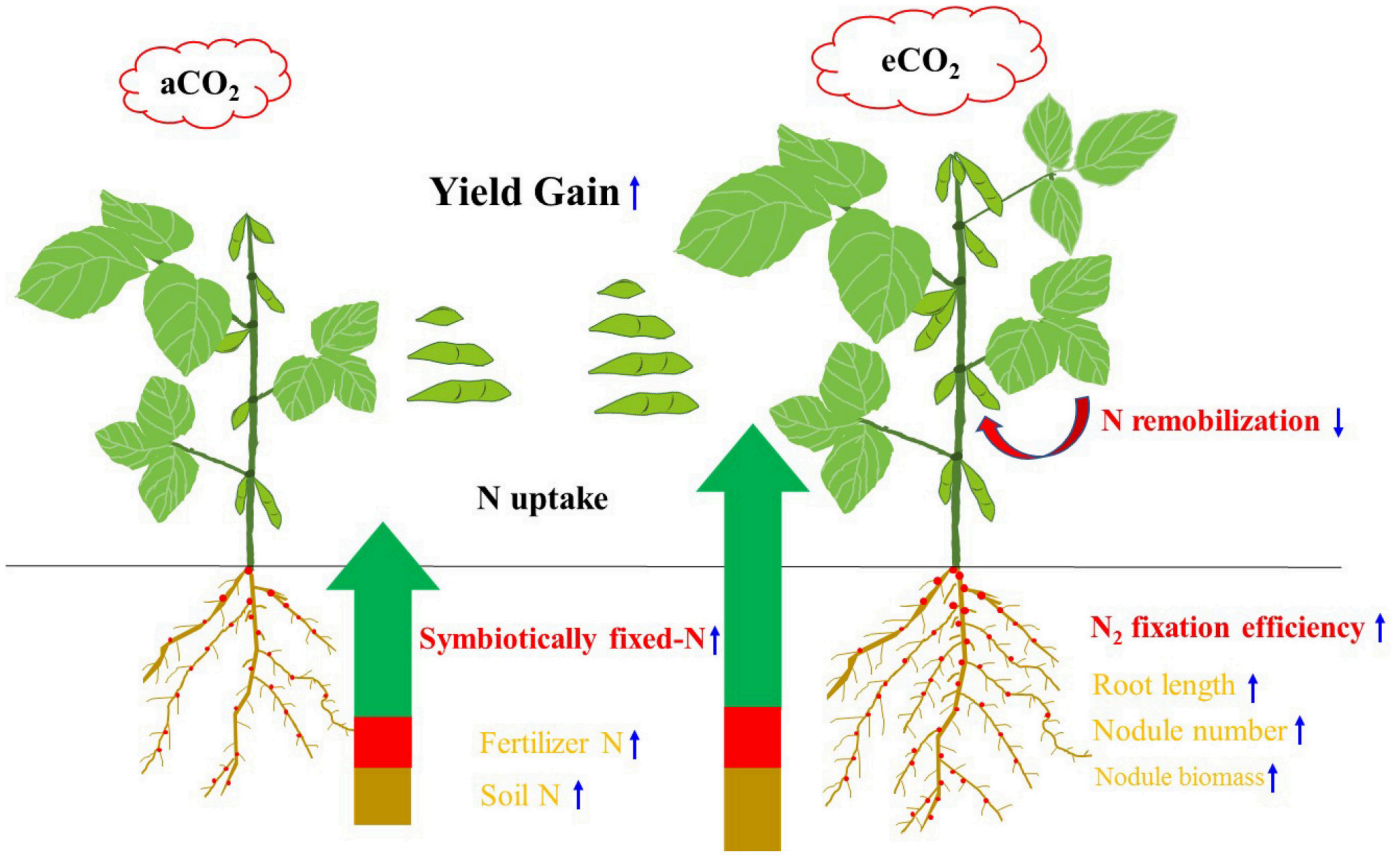
Diagram illustrating the N origins, root morphology, N remobilization, and yield gain of soybean in response to eCO_2_. The measurements that were significantly correlated with yield gain (*P* < 0.05) are indicated in red-bold, while the measurements responding to eCO_2_ but not correlated with yield gain are shown in orange. Upward and downward arrows indicate increase and decrease under the eCO_2_ condition, respectively.

In summary, Figure 7 shows a conceptual diagram illustrating how eCO_2_ affects N uptake, and consequent yield gain in soybean. Elevated CO_2_ increased the plants' ability for N uptake. The N_2_ fixation during R5 to R8 became a major contributor to the increased N uptake and hence yield gain under eCO_2_. The enhanced N_2_ fixation under eCO_2_ might also lead to the decrease in remobilization of N from vegetative organs, increasing photosynthetic capacity and yield formation. Although eCO_2_ facilitated root proliferation and nodule growth, these variables were not correlated with yield gains. Cultivars with a greater N_2_-fixing efficiency during the late reproductive phase may exhibit a better adaptability to eCO_2_. The specific interaction between cultivar and rhizobia in the rhizosphere of soybean would be the key to this adaptability, and is worth further investigation.

## Author contributions

JJ and YL designed the experiments and managed the projects. YL, ZY, JL, SZ, and JW performed experiments. YL, JJ, UM, GW, and CT performed data analysis. JJ, UM, YL, XL, and CT wrote the manuscript.

### Conflict of interest statement

The authors declare that the research was conducted in the absence of any commercial or financial relationships that could be construed as a potential conflict of interest.
