# Supplementary material for: Elevated CO2 Increases Nitrogen Fixation at the Reproductive Phase Contributing to Various Yield Responses of Soybean Cultivars
Source: Front Plant Sci. 2017 Sep 14;8:1546. doi: 10.3389/fpls.2017.01546 (PMC5603704; doi:10.3389/fpls.2017.01546)
Supplement: Supplementary file 1 [file DataSheet1.docx]

**Supplement figure and tables**

**Figure S1∣**The effect of eCO_2_ on shoot dry weight at (a) R5 (81 days after sowing) and (b) R8 (120 days after sowing), shoot N concentration at (c) R5 and (d) R8. Error bars are standard errors. The separate vertical bar in each panel indicates the LSD (*P* < 0.05) for the CO_2_ × cultivar interaction.

**Figure S2∣**Relationship between the increase in seed yield under eCO_2_ and increases in the amount of (a) fertilizer and (b) soil-derived N in shoot in response to eCO_2_. Each data point represents one cultivar.

**Figure S3∣**Relationships between the increase in seed yield under eCO_2_ and increases in (a) total root length, and length of (b) fine roots (0.0－0.5 mm in diameter), (c) intermediate roots (0.5－1.0 mm in diameter), and (d) coarse roots (>1.0 mm in diameter) in response to eCO_2_. Each data point represents one cultivar.

**Figure S4∣**Relationships between the increase in fixed-N content of shoot at R8 (120 days after sowing) under eCO_2_ and increases in (a) nodule number, (b) nodule density, (c) nodule fresh weight. Each data point represents one cultivar.

**Table S1∣**Root length among three root-diameter classes of eight soybean cultivars grown under aCO_2_ or eCO_2_ at R5 (81 days after sowing)

|  | Total root length  (m plant^-1^) | |  | | Fine roots length  (m plant^-1^) | |  | | Intermediate roots length  (m plant^-1^) | |  | | Coarse roots length  (m plant^-1^) | |  |
| --- | --- | --- | --- | --- | --- | --- | --- | --- | --- | --- | --- | --- | --- | --- | --- |
|  | aCO_2_ | eCO_2_ | | aCO_2_ | | eCO_2_ | | aCO_2_ | | eCO_2_ | | aCO_2_ | | eCO_2_ | |
| XHJ | 58.8 | 70.3^*^ | | 41.4 | | 49.8^*^ | | 11.5 | | 14.1^*^ | | 6.0 | | 6.4 ^ns^ | |
| SN14 | 48.7 | 59.9^*^ | | 34.8 | | 43.5^*^ | | 10.5 | | 13.5 ^ns^ | | 4.9 | | 5.6 ^ns^ | |
| SN8 | 63.6 | 74.0^*^ | | 45.4 | | 53.5^*^ | | 9.0 | | 10.8 ^ns^ | | 5.9 | | 7.0 ^ns^ | |
| HN45 | 55.5 | 61.0^*^ | | 39.8 | | 44.4^*^ | | 12.2 | | 13.6 ^ns^ | | 4.9 | | 5.3 ^ns^ | |
| SN22 | 64.3 | 66.4 ^ns^ | | 47.9 | | 46.9^ns^ | | 10.8 | | 11.3 ^ns^ | | 5.4 | | 6.6^*^ | |
| HJ6 | 52.5 | 72.8^*^ | | 37.1 | | 52.2^*^ | | 11.0 | | 12.8 ^ns^ | | 4.8 | | 7.2 ^ns^ | |
| NF9 | 51.9 | 59.8^*^ | | 37.1 | | 43.8^*^ | | 10.6 | | 10.6 ^ns^ | | 4.2 | | 5.5 ^ns^ | |
| NF1 | 57.1 | 62.3^*^ | | 40.2 | | 43.0^*^ | | 11.9 | | 12.2 ^ns^ | | 5.0 | | 7.1^*^ | |
| LSD_0.05_ | 4.46 | | | 3.28 | | | | 1.72 | | | | 1.81 | | | |
| *Significant level* |  |  | |  | |  | |  | |  | |  | |  | |
| CO_2_ | <0.001 | | | <0.001 | | | | <0.001 | | | | <0.001 | | | |
| Cultivar | <0.001 | | | <0.001 | | | | <0.001 | | | | 0.133 | | | |
| CO_2_×Cultivar | 0.001 | | | <0.001 | | | | 0.156 | | | | 0.697 | | | |

Fine roots (0.0－0.5 mm in diameter); Intermediate roots (0.5－1.0 mm in diameter); Coarse roots (>1.0 mm in diameter). * and ns indicate significant and non-significant differences (*t* test) between aCO_2_ and eCO_2_ of individual genotypes, respectively, for individual cultivars. LSD values correspond to the CO_2_ × cultivar interaction (two-way ANOVA).

**Table S2∣**Fertilizer- and soil-derived, and total N uptake per unit of root length for eight soybean cultivars grown for 120 days (R8) under aCO_2_ or eCO_2_

|  | N uptake by root  (mg m^-1^) | |  | | Fertilizer N uptake by root  (mg m^-1^) | |  | | Soil N uptake by root  (mg m^-1^) | |
| --- | --- | --- | --- | --- | --- | --- | --- | --- | --- | --- |
|  | aCO_2_ | eCO_2_ | | aCO_2_ | | eCO_2_ | | aCO_2_ | | eCO_2_ |
| XHJ | 3.03 | 3.93^*^ | | 1.95 | | 2.37 ^ns^ | | 1.08 | | 1.56^*^ |
| SN14 | 3.21 | 3.65^*^ | | 2.00 | | 2.12 ^ns^ | | 1.21 | | 1.53^*^ |
| SN8 | 3.14 | 3.07 ^ns^ | | 1.98 | | 1.80 ^ns^ | | 1.16 | | 1.27^*^ |
| HN45 | 3.29 | 3.73^*^ | | 2.09 | | 2.18 ^ns^ | | 1.2 | | 1.55^*^ |
| SN22 | 2.50 | 2.90^*^ | | 1.59 | | 1.70 ^ns^ | | 0.91 | | 1.20^*^ |
| HJ6 | 2.82 | 2.97 ^ns^ | | 1.76 | | 1.73 ^ns^ | | 1.06 | | 1.24^*^ |
| NF9 | 3.92 | 3.76 ^ns^ | | 2.47 | | 2.17^*^ | | 1.44 | | 1.58^*^ |
| NF1 | 2.69 | 3.34^*^ | | 1.68 | | 1.94^*^ | | 1.01 | | 1.41^*^ |
| LSD_0.05_ | 0.32 | | | 0.22 | | | | 0.11 | | |
| *Significant level* |  |  | |  | |  | |  | |  |
| CO_2_ | <0.001 | | | 0.106 | | | | <0.001 | | |
| Cultivar | <0.001 | | | <0.001 | | | | <0.001 | | |
| CO_2_×Cultivar | <0.001 | | | 0.001 | | | | <0.001 | | |

* and ns indicate significant and non-significant differences (*t* test) between aCO_2_ and eCO_2_, respectively, for individual cultivars. LSD values correspond to the CO_2_ × cultivar interaction (two-way ANOVA).
